# Effects of substitution of soybean meal with rapeseed meal and glutamine supplementation on growth performance, intestinal morphology, and intestinal mucosa barrier of Qiandongnan Xiaoxiang Chicken

**DOI:** 10.5713/ab.21.0467

**Published:** 2022-09-15

**Authors:** Bolin Zhang, Ning Liu, Meilin Hao, Yuxiao Xie, Peiyong Song

**Affiliations:** 1 Department of Biology and Agriculture, Characteristic Laboratory of Animal Resources Conservation and Utilization of Chishui River Basin, Zunyi Normal College, Hong Huagang, Zunyi 563006, China

**Keywords:** Glutamine, Intestinal Morphology, Qiandongnan Xiaoxiang Chicken, Rapeseed Meal, Tight Junction Protein

## Abstract

**Objective:**

The present study was to evaluate the effects of different rapeseed meal substitution (RSM) and glutamine (Gln) supplementation on growth performance, intestine morphology, and intestinal mucosa barrier of broilers.

**Methods:**

Four hundred and twenty Qiandongnan Xiaoxiang Chicken at 1 day of age with similar weight were chosen and were randomly assigned into 7 groups, consisting of 10 replicates per group and 6 broilers per replicate. Three groups were provided with diets separately containing 0%, 10%, and 20% RSM, and the other four groups were fed with diets separately supplemented with 0.5% and 1% Gln based on the inclusion of 10% and 20% RSM. At 21 and 42 days of age, 10 broilers per group were chosen to collect plasma and intestinal samples for further analysis.

**Results:**

The results showed that 10% RSM decreased average daily feed intake (ADFI) and average daily weight gain (ADG) of broilers at 21 days of age (p<0.05). Furthermore, both ADFI and ADG of broilers at 21 and 42 days of age were decreased by 20% RSM, while feed conversion ratio (FCR) was increased (p<0.05). Besides, 10% RSM resulted in lower intestinal villus height and the ratio of villus height to crypt depth, deeper crypt depth (p< 0.05), combined with the lower mRNA expressions of occludin, claudin-1, and zonula occludens-1 (ZO-1) in broilers at 21 days of age (p<0.05). Similar results were also observed in broilers at 21 and 42 days of age fed with 20% RSM. However, 1% Gln improved the growth performance of broilers fed with 10% and 20% RSM (p<0.05), ameliorated intestine morphology and elevated mRNA expressions of occludin, claudin-1 and ZO-1 (p<0.05).

**Conclusion:**

In conclusion, the increasing inclusion of RSM resulted in more serious effects on broilers, however, 1.0% Gln could reverse the negative effects induced by the inclusion of RSM.

## INTRODUCTION

Soybean meal is traditionally used as a major vegetable protein source in broiler diets [1]. However, owing to the rising price of soybean and its products, it is an important consideration to searching for properly substitute of soybean protein. Rapeseed meal (RSM), which is composed of 35% to 42% crude protein and is a good protein ingredient in animal feed for its well-balanced amino acid and higher contents sulfur-containing amino acids, can be a valuable source of energy and protein for poultry [2–4]. But the presence of anti-nutrition factors in RSM, such as glucosinolates and other anti-nutritional ingredients, limit its usage in broiler diets. Although accumulated evidences on the effects of RSM addition on the growth performance of broilers are available [5,6], the results of which are inconsistent. In a previous study, it was reported that no negative effect on the growth performance of Cobb-500 broilers from 1–42 days of age was observed in response to the inclusion of RSM in diet up to 10% [6]. In contrast, McNeill et al [7] found that the inclusion of 10% and 20% RSM in diet resulted in a decrease in feed intake and weight gain of Ross broilers from 1 to 42 days, but the feed conversion ratio (FCR) was affected only from 1 to 21 days. The contradictions on the responses of broilers to dietary RSM inclusion may be attributed to the breed and age of broilers being reared, the processing of RSM, diet composition and feed processing treatments [8]. Moreover, RSM has been used extensively to reduce the feed costs of broilers in China, but a proper incorporation of RSM in the native broiler breeds is still unclear.

If alternative to soybean meal protein supplements are proposed for use in animal diets, it is crucial to consider the effects of these feed ingredients on the intestinal mucosa [9]. Hu et al [10] reported that, compared to the control group (without no RSM supplementation), 10.9% RSM supplementation decreased the villus height (VH) of duodenum, jejunum and ileum of broilers at 42 days of age. Similarly, a previous study conducted by Gopinger et al [9] found that the decreased VH of duodenum of broilers occurred as the inclusion of RSM up to 20% or beyond this level. The results mentioned above indicated that RSM supplementation at a certain level in diet may damage the small intestinal morphology of broilers. Nevertheless, the small intestine is the primary organ responsible for the digestion, hydrolysis and absorption of nutrients and mucosal barrier functions. Therefore, the sooner the small intestine achieves its functional capacity, the sooner the broiler can utilize dietary nutrients and grow according to its genetic potential and then resist infectious or metabolic disease [11,12]. Therefore, it is necessary to conduct nutritional modulation, such as exogenous active substances addition, to protect the intestinal health to ensure the maximum growth of broilers fed with RSM.

It has been demonstrated that glutamine (Gln) is a principal metabolic fuel for small intestine enterocytes and plays a vital role in promoting the growth and development of the gastrointestinal tract [13]. Among the various tissues using Gln at high rates, the intestine can utilize about 30% of total Gln, indicating that it is a key nutrient for the intestine [14]. Bartell and Batal [15] found that 1% Gln supplementation resulted in an increase in the intestinal VH of duodenum and jejunum. Moreover, similar results were also observed in a previous study conducted by Wu et al [16]. Besides, Gln supplementation was proven to maintain the intestinal tissue integrity and enhance the small intestine mucosa barrier [17]. However, up to now, no information has been found in the available literatures concerning the effects of Gln supplementation on the growth performance, small intestine development and intestinal epithelial barrier of broilers fed with RSM.

Therefore, the objectives of the present study were to test the hypothesis that dietary supplemented with Gln could elevate the growth performance, improve small intestine morphology and enhance the intestine mucosa barrier of broilers fed with different levels of RSM.

## MATERIALS AND METHODS

### Animal care and management

All the procedures including animal care and experiment treatments in the present study were conducted in accordance with guidelines concerning the animal welfare formulated by the Institutional Animal Care and Use Committee of Zunyi Normal College.

In total, four hundred and twenty male Qiandongnan Xiaoxiang Chicken of newly-hatched were obtained from a commercial hatchery (Rongjiang Shannong Development Co., Ltd, Guizhou, China). The newly-hatched broilers with similar weight were randomly assigned into 7 groups, with 10 replicates per group and 6 chicken per replicate. Three groups were provided with diets separately containing 0%, 10%, and 20% RSM, and the other four groups were fed with diets separately supplemented with 0.5% and 1% Gln based on the inclusion of 10% and 20% RSM. The concentration of Gln supplemented in the diet was fully exogenous. All diets were formulated to meet or exceed the recommendations of feeding standard of chicken. The raw materials of Gln were purchased from Shanghai Feiya Technology Development Co., Ltd. (Shanghai, China). The diet composition and nutrients level are shown in Table 1.

During the entire experiment, broilers were raised in three-level cages (120 cm×80 cm×60 cm) and placed a temperature-controlled room. In the first three days, room temperature was kept at 34°C to 36°C and were gradually decreased by 2°C to 3°C weekly until reaching 24°C to 26°C, which was maintained to the end of the experiment. The light procedure was 23 h light and 1h dark and the humidity of varied from 60% to 65% during the experimental period. Broilers were freely access to feed in mash form and water *ad libitum*. The duration of the experiment was 42 days. Feed consumption per cage were recorded daily and broilers were weighted at 21 d and 42 d after 8 h feed deprivation without water withdraw.

### Sampling

At 21 d and 42 d of age, 10 broilers from each group were randomly selected for blood sampling via wing vein puncture. Blood samples were collected into tubes containing ethylenediamine tetraacetate disodium and were centrifuged at 4°C, 3,000×g for 10 min to collect supernatants for further analysis. Immediately after blood sampling, the selected broilers were sacrificed by cervical dislocation followed by exsanguination. The abdominal and thoracic cavities were then opened and the duodenum (from the end of the pylorus to the end of the pancreatic loop), the jejunum (from the end of the pancreatic loop to Meckel’s diverticulum) and ileum (from Meckel’s diverticulum to the cecal junction) were immediately collected and emptied using gentle pressure. The dissected small intestine was placed on a chilled stainless steel tray. About 3 to 5 cm segments of mid-jejunum and mid-ileum were removed, opened longitudinally, flushed with ice-cold PBS buffer (pH 7.4) and fixed in the chilled neutral-buffered formalin solution for subsequent gut histological measurement. The remaining portion of the duodenum, jejunum and ileum were excised, mucosa samples were scraped into 2 mL sterile tubes using glass microscope slides and immediately frozen in liquid nitrogen until further analysis. All samples were collected within 10 minutes after killing.

### Growth performance

At 21 d and 42 d of age, broilers were weighed after an 8 h feed deprivation without water withdraw to avoid weight errors induced by different feed intakes. The total feed consumption and the total weight of birds for each cage were separately recorded to calculate average daily feed intake (ADFI), average daily gain (ADG), and the ratio of feed to gain.

### Plasma parameters

The concentrations of total protein (TP) and glucose (Glu) in plasma were assayed with commercially available kits (Nanjing Jiancheng Bioengineering Institute, Nanjing, China). The procedure was conducted according to the manufacture’s instructions.

### Intestinal morphology measurements

The intestine segments for morphological measurements were dehydrated through a graded series of alcohols (50% alcohol, 2 h; 75% alcohol, 2 h; 85% alcohol, 2 h; 95% alcohol, 30 min; 95% alcohol, 30 min; anhydrous alcohol, 20 min and anhydrous alcohol, 20 min) and embedded in paraffin, then were cut into serial sections of 5 μm thickness and stained with hematoxylin and eosin for light microscopy examination. Villus height was measured from the tip of villus to the junction of villus and crypt. Crypt depth (CD) was defined as the depth of invagination between adjacent villi. Villus height and CD were determined using Image-Pro Plus 6.0. Ten well-oriented, intact villi-crypts units were selected in triplicate for each section.

### Total RNA extraction and real-time polymerase chain reaction measurement

Total RNA extracted from duodenum, jejunum and ileum samples were conducted using RNAiso Plus reagent (catalogue no. 9108, TaKaRa Biotechnology (Dalian) CO., LTD. Dalian, China). The purity of the isolated RNA was quantified by evaluating the OD_260_/OD_280_ ratio with a ND-1000 spectrophotometer (NanoDrop; Thermo Fisher Scientific, Waltham, MA, USA). Subsequently, reverse transcription and real-time polymerase chain reaction (Real time RT-PCR) were performed using the PrimeScriptTM RT Master Mix (catalogue no. RR037A; TaKaRa Biomedical Technology Co., Ltd., China) and TB Green Premix Ex Taq (catalogue no. RR420A; TaKaRa Biomedical Technology Co., Ltd., China), respectively. All procedures were conducted in accordance with the instructions of manufacture. PCR program consisted of one cycle at 95°C for 30 s, 40 cycles of denaturation at 95°C for 5 s, and a 60°C annealing step for 30 s. Melting curve was performed to verify the specificity of PCR amplified product. The expression of target genes relative to the housekeeping gene (*β-actin*) were calculated by 2^−ΔΔCt^ method [18]. All samples were handled in triplicate. The primer sequences for the target and housekeeping genes are shown in Table 2.

### Statistical analysis

Data were analyzed by one-way analysis of variance (ANOVA) of SPSS (SPSS version 20.0) and differences between means were further determined by Duncan’s multiple comparison test. Besides, all data except for the control group were analyzed by a 2×3 factorial arrangement (SPSS version 20.0). The statistical model consisted of the effects of RSM inclusion (10% or 20%) and Gln supplementation (0%, 0.5%, or 1%) and their interactions. The pen was as an experimental unit for growth performance, whereas the individual broiler was the experimental unit for the other parameters (n = 10). Statistical significance is defined when p values are less than 0.05. All values are shown as means and standard error of the mean.

## RESULTS

### Growth performance

As shown in Table 3, the main effects analysis results showed that there were no interactions between RSM and Gln on the growth performance of broilers at 21 and 42 days of age (p>0.05). The one-way ANOVA results showed that, compared with the control group, 10% RSM inclusion significantly decreased the ADFI and ADG of broilers at 21 days of age (p<0.05). But there was no difference in FCR of broilers at 21 days of age (p>0.05). Moreover, there were also no differences in ADFI, ADG, or FCR of broilers at 42 days of age fed with 10% RSM diet compared with those fed with the control diet (p>0.05). However, in comparison with the control group, both ADFI and ADG of broilers at 21 and 42 days of age were significantly decreased by 20% RSM inclusion, while FCR was significantly increased (p<0.05). Furthermore, for broilers at 21 and 42 days of age, a significant decrease in ADG and a significant increase in FCR were observed with increasing of the inclusion of RSM (p<0.05). Compared with broilers fed with diet containing 10% RSM, 1% Gln supplementation increased ADFI and ADG of broilers at 21 and 42 days of age (p<0.05). However, not 42 days old but 21 days old broilers in FCR was decreased by 1% Gln supplementation based on 10% RSM inclusion (p<0.05). In contrast, 1% Gln supplementation resulted in higher ADG and ADFI and lower FCR of broilers at 21 and 42 days of age compared with the control group (p<0.05). For those fed with diet containing 20% RSM, 1% Gln supplementation increased ADFI and ADG at 21 and 42 days of age (p<0.05), and decreased FCR (p<0.05). But there were no differences in ADFI, ADG, or FCR of broilers at 21 and 42 days of age between 1% Gln supplementation based on 20% RSM inclusion and the control group (p>0.05).

### Biochemical parameters

The main effect analysis results showed that there were no interactions between RSM and Gln on the concentration of TP and Glu of broilers at 21 and 42 days old (p>0.05, Table 4). The one-way ANOVA results showed that both 10% and 20% RSM inclusion did not affect the concentration of Glu in plasma of broilers at 21 and 42 days of age compared with those in the control group (p>0.05). Compared with the control group, 20% RSM inclusion significantly decreased the concentration of TP of broilers at 21 and 42 days of age (p<0.05), however, the concentration of TP was not affected by 10% RSM inclusion (p>0.05). Based on the supplementation with 10% and 20% RSM, 1% Gln addition increased the concentration of TP of broilers at 21 days of age and the contents of TP of broilers at 42 days of age (p<0.05).

### Intestinal morphology of jejunum

**Figure 1:**
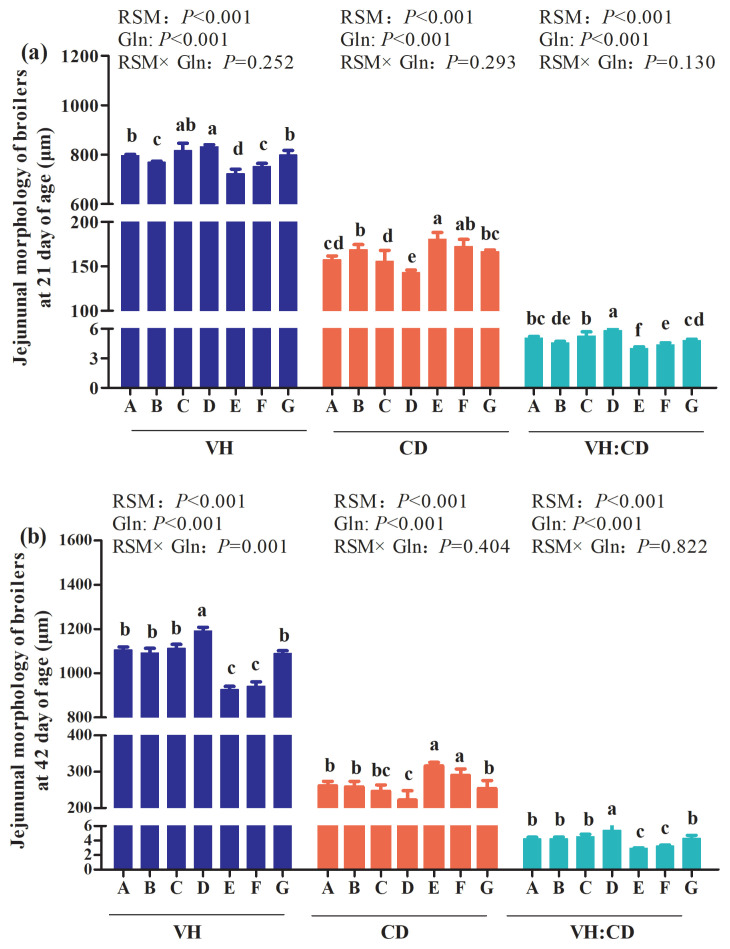
Effects of different levels of rapeseed meal substitution and glutamine on jejunum morphology of Qiandongnan Xiaoxiang Chicken at 21 and 42 days of age (n = 10 per treatment group). Columns in the same color with different small letters superscripts mean significant differences (p<0.05). Broilers in the control group (A) were fed with basal diet. Broilers in 10% (B) and 20% RSM (E) were fed with diet containing 10% or 20% RSM, respectively. 10% RSM+0.5% Gln (C), 10% RSM+1.0% Gln (D), 20% RSM+0.5% Gln (F), and 20% RSM+1.0% Gln (G) mean that 0.5% Gln or 1% Gln was separately supplemented based on 10% and 20% RSM inclusion.

As shown in Figure 1, the main effect of analysis results showed that there were no interactions between RSM and Gln on jejunum morphology of broilers at 21 and 42 days of age (p>0.05), except for jejunum VH of broilers at 42 days of age (p = 0.001). The main effect of analysis indicated that RSM supplementation decreased VH of jejunum in broilers at 42 days of age (p<0.001) and Gln supplementation reversed the negative effects induced by RSM addition (p<0.001). The one-way ANOVA results showed that, compared with the control group, both 10% RSM and 20% RSM inclusion significantly decreased jejunum VH and VH:CD, increased jejunum CD of Qiandongnan Xiaoxiang Chicken at 21 days of age (Figure 1(a); p<0.05). Similar results were also observed in broilers at 42 days of age, which were fed with diet containing 20% RSM (Figure 1(b); p<0.05). However, compared with the control group, VH, CD, and VH:CD of broilers at 42 days of age were not affected by 10% RSM inclusion (p> 0.05). In comparison with broilers at 21 days of age, which were fed with diet containing 10% RSM or 20% RSM, the significant increased VH and VH:CD, combined with the decreased CD were observed in response to 0.5% and 1% Gln supplementation (p<0.05), except for jejunum CD of broilers fed with 20% RSM diet (p>0.05). Besides, compared with broilers fed with 10% or 20% RSM diet, 1% Gln supplementation significantly increased VH and VH:CD and decreased CD of broilers at 42 days of age fed with 10% RSM or 20% RSM diet (p<0.05). In contrast, in comparison with 10% RSM or 20% RSM inclusion, there were no differences in VH, CD, or VH:CD of broilers by 0.5% Gln supplementation based on 10% or 20% RSM addition (p>0.05). Compared with the control group, broilers fed with the diet containing 1% Gln showed a significant increase of VH and VH:CD, and a decrease of CD at 21 days of age based on 10% RSM inclusion (p<0.05). However, 1% Gln addition did not influence VH, CD, or VH:CD of broilers at 42 days of age fed with 20% RSM diet when compared with those in the control group (p>0.05).

### Intestinal morphology of ileum

**Figure 2:**
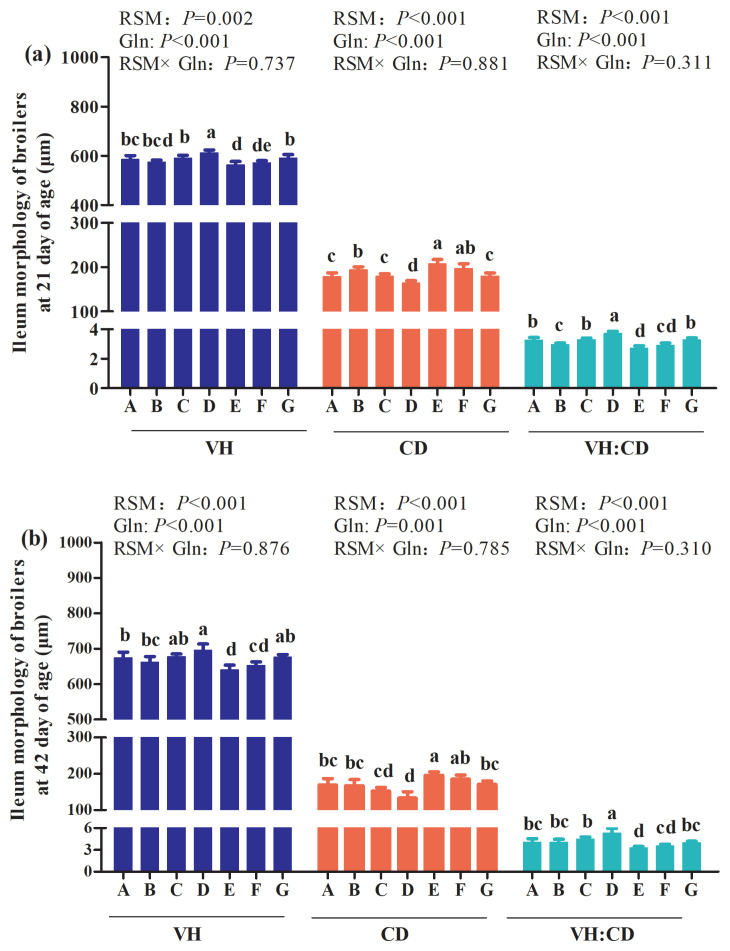
Effects of different levels of rapeseed meal substitution and glutamine on ileum morphology of Qiandongnan Xiaoxiang Chicken at 21 and 42 days of age (n = 10 per treatment group). Columns in the same color with different small letters superscripts mean significant differences (p<0.05). Broilers in the control group (A) were fed with basal diet. Broilers in 10% (B) and 20% RSM (E) were fed with diet containing 10% or 20% RSM, respectively. 10% RSM+0.5% Gln (C), 10% RSM+1.0% Gln (D), 20% RSM+0.5% Gln (F), and 20% RSM+1.0% Gln (G) mean that 0.5% Gln or 1% Gln was separately supplemented based on 10% and 20% RSM inclusion.

The main effect analysis results showed that no interactions between RSM and Gln supplementation on ileum morphology of broilers at 21 and 42 days of age were observed (p> 0.05; Figure 2). However, the one-way ANOVA results showed that, compared with the control group, 20% RSM inclusion significantly decreased ileum VH and VH:CD, and increased CD of Qiandongnan Xiaoxiang Chicken at 21 (Figure 2(a)) and 42 days (Figure 2(b)) of age (p<0.05). Nevertheless, compared with the control group, there were no differences in ileum VH, CD or VH:CD in broilers at 21 and 42 days of age fed with 10% RSM diet (p>0.05), except for the increased CD and decreased VH:CD of broilers at 21 days of age (p< 0.05). In comparison with 10% RSM or 20% RSM inclusion, the broilers at 21 and 42 days of age both exhibited a significant increase in VH and VH:CD, and a decrease in CD in response to 1% Gln supplementation (p<0.05). However, 0.5% Gln supplementation merely decreased ileum CD and increased VH:CD of broilers at 21 days of age (p>0.05) when compared with those fed with diet containing 10% RSM or 20% RSM (p>0.05). The VH and VH:CD of ileum were increased while CD was decreased by 1% Gln supplementation at 21 and 42 days of age fed with 10% RSM diet when compared with the control group (p<0.05). In contrast, in comparison with the control group, 1% Gln supplementation did not affect the ileum VH, CD, or VH:CD of broilers fed with the diet containing 20% RSM (p>0.05).

### mRNA expressions of intestine tight junction protein of broilers at 21 days of age

**Figure 3:**
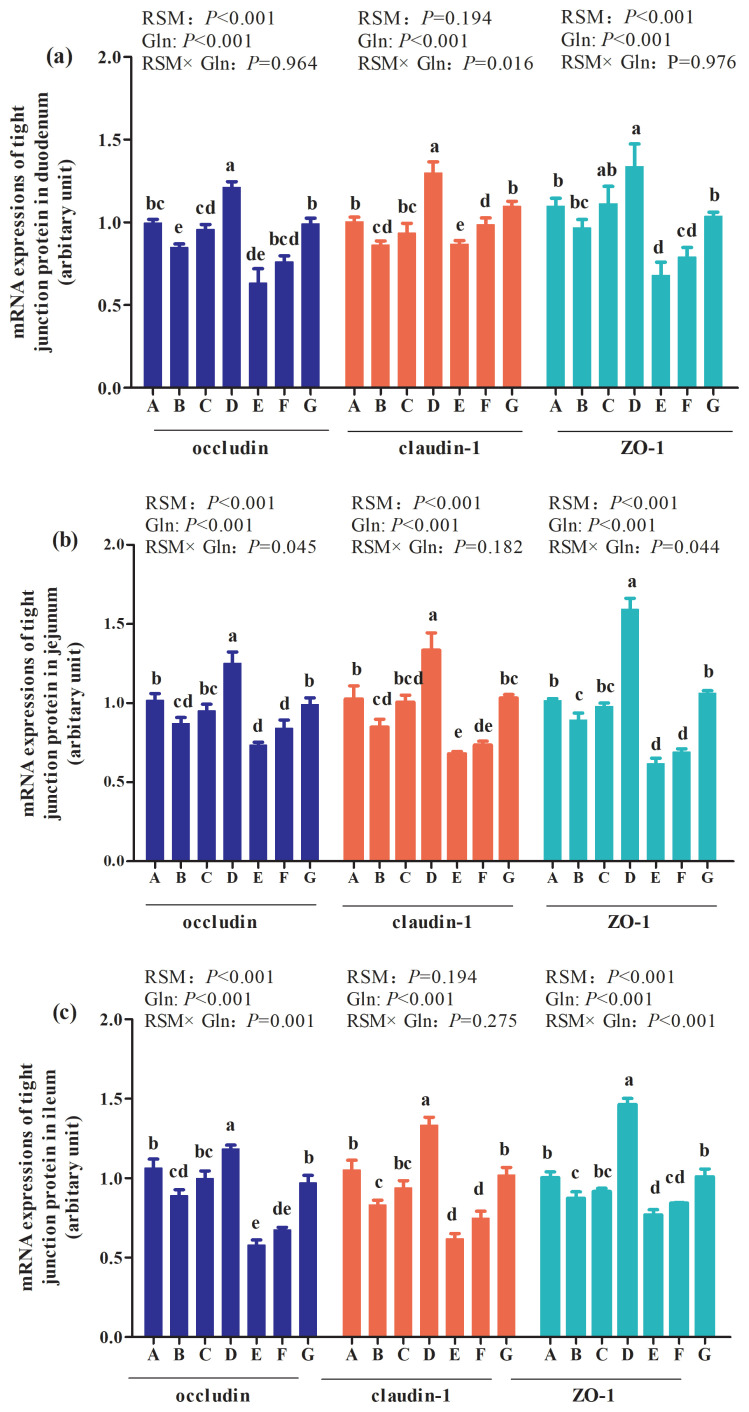
Effects of different levels of rapeseed meal substitution and glutamine on mRNA expression of intestinal tight junction protein of Qiandongnan Xiaoxiang Chicken at 21 days of age (n = 10 per treatment group). Columns in the same color with different small letters superscripts mean significant differences (p<0.05). Broilers in the control group (A) were fed with basal diet. Broilers in 10% (B) and 20% RSM (E) were fed with diet containing 10% or 20% RSM, respectively. 10% RSM+0.5% Gln (C), 10% RSM+1.0% Gln (D), 20% RSM+0.5% Gln (F), and 20% RSM+1.0% Gln (G) mean that 0.5% Gln or 1% Gln was separately supplemented based on 10% and 20% RSM inclusion.

As presented in Figure 3, the main effect of analysis results showed that there were interactions between RSM and Gln supplementation on claudin-1 in duodenum (p = 0.016; Figure 3(a)), combined with occludin (p = 0.045; Figure 3(b)) and occludens-1 (ZO-1) (p = 0.044; Figure 3(c)) in jejunum of broilers at 21 days of age. Moreover, an interaction between RSM and Gln supplementation on occludin (p = 0.001) and ZO-1 (p<0.001) in ileum of broilers at 21 days of age was also observed. But there were no interactions on other tight junction proteins of duodenum, jejunum or ileum of broilers at 21 days of age (p>0.05). The one-way ANOVA results showed that, compared with the control group, 10% RSM inclusion decreased mRNA expressions of claudin-1, occludin, and ZO-1 in the mucosa of duodenum, jejunum and ileum of broilers (p<0.05), with the exception of duodenum ZO-1 mRNA expression of broilers at 21 days of age (p>0.05). Moreover, 20% RSM inclusion decreased mRNA expressions of claudin-1, occludin, and ZO-1 in mucosa of duodenum, jejunum and ileum of broilers in comparison with those in the control group (p<0.05). Besides, 1% Gln supplementation increased mRNA expressions of claudin-1, occluding, and ZO-1 in duodenum, jejunum and ileum of broilers fed with 10% RSM or 20% RSM diet (p<0.05). However, there were no differences in mRNA expressions of claudin-1, occludin, or ZO-1 in mucosa of duodenum, jejunum and ileum of broilers supplemented with 0.5% Gln when compared with those fed with 10% RSM diet (p>0.05), except for the increased occludin mRNA expression in duodenum of broiler supplemented with 10% RSM diet (p<0.05). Compared with those fed with diet containing 20% RSM, an increase in occludin mRNA expression in duodenum was observed by 0.5% Gln supplementation (p<0.05). Compared with the control group, higher mRNA expressions of claudin-1, occludin, and ZO-1 in mucosa of duodenum, jejunum and ileum of broilers fed with 10% RSM diet were observed by 1% Gln supplementation (p<0.05). Nevertheless, in comparison with those in the control group, 1% Gln addition did not affect mRNA expressions of claudin-1, occludin, or ZO-1 in duodenum, jejunum and ileum of mucosa of broilers fed with 20% RSM diet (p>0.05).

### mRNA expressions of intestine tight junction protein of broilers at 42 days of age

**Figure 4:**
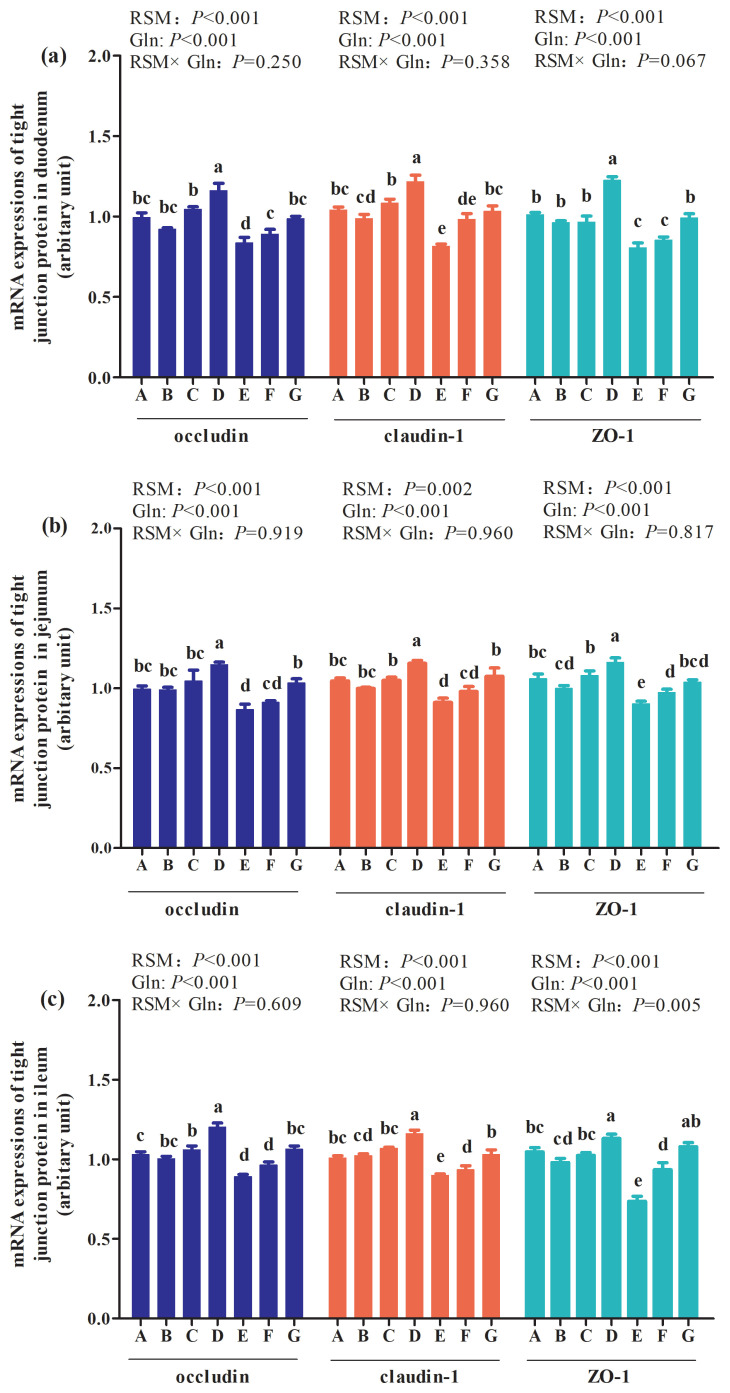
Effects of different levels of rapeseed meal substitution and glutamine on mRNA expression of intestinal tight junction protein of Qiandongnan Xiaoxiang Chicken at 42 days of age (n = 10 per treatment group). Columns in the same color with different small letters superscripts mean significant differences (p<0.05). Broilers in the control group (A) were fed with basal diet. Broilers in 10% (B) and 20% RSM (E) were fed with diet containing 10% or 20% RSM, respectively. 10% RSM+0.5% Gln (C), 10% RSM+1.0% Gln (D), 20% RSM+0.5% Gln (F), and 20% RSM+1.0% Gln (G) mean that 0.5% Gln or 1% Gln was separately supplemented based on 10% and 20% RSM inclusion.

As shown in Figure 4, the main effect analysis results showed that there was an interaction between RSM and Gln supplementation on ZO-1 mRNA expressions (p = 0.005; Figure 4(c)) in ileum of broilers at 42 days of age. Besides, an interaction trend occurred in duodenum ZO-1 mRNA expressions (p = 0.065; Figure 4(a)), and no other interactions were observed on mRNA expressions of tight junction proteins in duodenum, jejunum and ileum of broilers at 42 days of age (p>0.05). The one-way ANOVA results showed that 20% RSM inclusion decreased mRNA expressions of claudin-1, occludin, and ZO-1 in the mucosa of duodenum, jejunum (Figure 4(b)) and ileum of broilers compared with the control group (p<0.05), however, there were no differences in mRNA expressions of genes mentioned above between the control group and 10% RSM group (p>0.05). In addition, mRNA expressions of claudin-1, occludin, and ZO-1 in duodenum, jejunum and ileum of broilers by 1% Gln supplementation were higher than those fed with 10% RSM or 20% RSM diet (p<0.05). However, 0.5% Gln supplementation increased duodenum claudin-1 and jejunum ZO-1 mRNA expressions of broiler fed with 10% RSM diet (p< 0.05), while 0.5% Gln addition also elevated mRNA expressions of duodenum occludin, jejunum ZO-1 and ileum claudin-1 and ZO-1 of broiler supplemented with 20% RSM diet (p<0.05). The mRNA levels of claudin-1, occludin, and ZO-1 in mucosa of duodenum, jejunum and ileum of broilers fed with 10% RSM diet were elevated by 1% Gln supplementation when compared with those in the control group (p< 0.05). But no differences in mRNA expressions of claudin-1, occluding, or ZO-1 in duodenum, jejunum and ileum of mucosa of broilers fed with 20% RSM diet were observed between 1% Gln supplementation and the control group (p>0.05).

## DISCUSSION

In previous studies, it was reported that 10% RSM inclusion did not affect the growth performance of broilers in the earlier growth period [9,19]. Besides, Rabie et al [6] found that 10% rapeseed meal replacement did not affect the growth performance of broilers. But in our present study, in the period of 1 to 21 d, dietary inclusion of 10% RSM showed a statistically significant effect on ADFI and ADG of broilers. In consistent with the results of ours, McNeill et al [7] also suggested that the inclusion of 10% RSM in the diet results in a significant decrease in feed intake and weight gain of broilers. The reduction of the growth performance of broilers may be associated with the fact that Qiandongnan Xiaoxiang Chicken is a native small chicken breed with slow-growing rate, its average body weight was only 125.7 g at 21 days of age, however, the average body weight of broilers used in the study of Rabie et al [6] was 715.8 g. Therefore, it could be concluded that the development of Qiandongnan Xiaoxiang Chicken was slower and immature, resulting in more susceptible to rapeseed meal. Moreover, 20% RSM inclusion exhibited negative effects on the growth performance of broilers at 21 and 42 days of age, evidenced by lower ADFI, ADG, and higher FCR. In our previous study, it demonstrated that the inclusion RSM up to 20% decreased feed intake and body weight [20]. Similar with the results of ours, a report conducted by Sina et al [21] found that the body weight of broilers was impaired with the increasing inclusion of rapeseed meal up to 20%. Min et al [22] also reported that the body weight of gain of broilers dramatically declined in response to the increasing inclusion of rapeseed meal from 0% to 25%. Nevertheless, whether for 10% RSM or 20% RSM, broilers at 21 or 42 days of age supplemented with 1% Gln had higher ADG, ADFI, and lower FCR, indicating that 1% Gln reversed the impaired effects induced by the inclusion of 10% and 20% RSM. Similar with the results of a previous study, it has been demonstrated that the growth performance of broilers is improved by 1% Gln supplementation, evidenced by the increased ADFI and ADG and the decreased FCR [23]. Xue et al [24] also reported that 1% Gln supplementation increased ADFI and ADG, and decreased FCR of broilers. Therefore, the results mentioned above indicated that 1% Gln supplementation could contribute to ameliorating the adverse effects of the inclusion of 10% and 20% RSM on the growth performance of broilers. It has been demonstrated that 1% Gln supplementation contributed to increasing feed efficiency

Blood parameters is directly related to the metabolic state of broilers and could indicate the function and metabolism of nutrients. TP content in plasma, to a certain extent, reflects the nutritional level of dietary protein and the synthesis of protein in the body. In the current study, the inclusion of 10% RSM had no effect on the concentration of TP. In consistent with the results of ours, Hu et al [10] found that 10.6% rapeseed meal supplementation did not affect the TP contents of broilers. But the inclusion of 20% RSM in diet resulted in the decreased TP content of broilers at 21 and 42 days of age, indicating that the digestion and metabolism of broilers were influenced with the increasing of RSM up to 20%. The concentration of TP in plasma of broilers fed with diets containing 10% and 20% RSM were increased by 1% Gln supplementation. In consistent with the results of ours, a previous study showed that 1% Gln supplementation increased TP contents in the plasma of Arbor Acres broilers [25]. Therefore, it could be concluded that 1% Gln supplementation could improve nutrition metabolism of Qiandongnan Xiaoxiang Chicken fed with diet containing RSM.

The small intestine is the main site responsible for nutrient digestion and absorption and the growth of broilers is associated with the morphological and functional integrity of the digestive system [9]. The values of VH, CD, and VH:CD of the small intestine are the useful indicators to estimate the absorption capacity of the small intestine [10,26]. In our present study, decreased VH and VH:CD, combined with the increased CD in jejunum and ileum were observed in broilers fed the diet containing 10% RSM of broilers at 21 days of age and broilers receiving the diet including 20% RSM of broilers both at 21 and 42 days of age. Chiang et al [26] found that the inclusion of 10% level of unfermented rapeseed meal did not have any adverse effects on the intestinal morphology except for the decreased VH of ileum in broilers at 21 d and the increased ratio of VH:CD in broilers at 42 d. Gopinger et al [9] suggested that the morphology of broilers maybe impaired with the increasing of the rapeseed meal to or above 20%. The depression of the small intestine induced by the inclusion of RSM could be associated with the anti-nutrition factors, fiber contents in the rapeseed meal [20]. Besides, the negative effects of the inclusion of 10% and 20% RSM on VH, CD, and VH:CD in both jejunum and ileum of broilers may be resulted in the impairment of the growth performance, which are in consistent with the results of the growth performance in our present study. However, in the present study, broilers receiving diets supplemented with 1% Gln had longer VH, lower CD, and higher VH:CD. To the best of our knowledge, up to now, few studies has been performed to determine the effects of Gln supplementation on intestine morphology of broilers fed with different levels of rapeseed meal. But it was reported that 1% Gln supplementation contributed to improving intestine morphology of broilers in the state of heat stress or necrotic enteritis challenge [16,24]. Moreover, it has been demonstrated that Gln can provide metabolic fuel for intestinal epithelial cells, promote cell proliferation and differentiation, and thus promote intestinal morphology and mucosal development [24]. Thus, the results mentioned above indicated that Gln may contribute to improving the adverse effects of high inclusion of RSM on intestine morphology.

It was demonstrated that the improvement of intestinal morphology including elevated VH, decreased CD, coupled with an increased the ratio of VH:CD are helpful to stress resistance and gut barrier functions of animals [27]. Intestinal epithelial cells are not only responsible for the nutrients digestion and absorption but also play an important role in regulating the intestinal mucosal barrier function [28]. The paracellular permeability of the intestinal barrier is regulated by a complex protein system called tight junctions [29]. Tight junction proteins, located at the most apical region of epithelial cells, serve as a molecular fence partitioning the cytosolic membrane into apical and basolateral domains. It has been demonstrated that tight junctions are consisted of more than 40 different proteins including occluding, claudin, and ZO-1 [30,31]. Indeed, in the present study, the mRNA expressions of ZO-1, claudin-1, and occludin in duodenum, jejunum and ileum were downregulated by 10% RSM of broilers at 21 days of age and the inclusion of 20% RSM of broilers at 21 and 42 days of age. It was reported that the inclusion 20% RSM resulted in the decreased of secretory immunoglobulin A in jejunum of Qiandongnan Xiaoxiang Chicken at 42 days of age, the main immune effector produced by plasma cells of intestinal mucosa and is the first defense of intestinal immune barrier [20]. This indicated that intestinal mucosa barrier was ruined by the inclusion of 20% RSM. However, 1% Gln supplementation improved intestinal mucosa barrier. It has been established that Gln are involved in the regulation of gut tight junction proteins [32]. It has been demonstrated that Gln is an important amino acid for maintenance of gut metabolism, structure, and function especially when the gut mucosal barrier compromised [33]. Restoration of tight junctions has been reported by Gln addition both *in vivo* [34] and *in vitro* [35]. Moreover, it was found that Gln deprivation in intestine epithelial cells was associated with a loss of tight junction proteins, whereas Gln addition rescued the phenotype of barrier dysfunction [36]. The protective effect of Gln on alleviating intestinal lesions may also be associated with the enhanced development of the intestinal mucosa [24]. The results mentioned above indicated that Gln may contribute to preserving the intestinal barrier function of broilers fed with RSM and furtherly improving the intestinal permeability.

## CONCLUSION

In conclusion, 10% RSM and 1% Gln combination supplementation in diets did not result in negative effects on Qiandongnan Xiaoxiang Chicken at 1 to 21 days of age. And the inclusion of 10% RSM in diet of Qiandongnan Xiaoxiang Chicken at 21 to 42 days of age was recommended. But the inclusion of RSM up to 20% in diets resulted in the impaired growth performance, the destruction of intestinal morphology and the damage of intestinal tight junction protein of Qiandongnan Xiaoxiang Chicken at 1 to 21 and 21 to 42 days of age. However, 1% Gln administration reversed the deleterious effects of RSM on broilers, evidenced by the improved growth performance, ameliorated of intestinal morphology and tight junction protein.

## Figures and Tables

**Table 1:** The ingredients and nutritional levels of diets

Items	Basal diet	10% RSM	20% RSM
Ingredient (%)			
Maize	56.30	56.30	56.30
Wheat bran	5.25	2.94	0.77
Corn gluten meal	4.35	6.33	8.23
Soybean meal	28.50	18.52	8.50
Rapeseed meal	0.00	10.00	20.00
Limestone	1.26	1.18	1.08
Sodium chloride	0.15	0.15	0.15
Dicalcium phosphate	1.87	1.89	1.95
Phytase	0.04	0.04	0.04
Choline chloride	0.10	0.15	0.10
Methionine	0.15	0.15	0.07
Lysine	0.00	0.22	0.45
Soybean oil	1.53	1.63	1.86
Premix^1)^	0.50	0.50	0.50
Total	100.00	100.00	100.00
Nutrient level			
Crude protein^2)^ (%)	21.13	21.18	21.10
Metabolizable energy (MJ/kg)	12.14	12.12	12.15
Calcium (%)	1.00	1.00	1.01
Available phosphorus (%)	0.46	0.45	0.46
Methionine+cystine^3)^ (%)	0.86	0.90	0.86
Lysine^3)^ (%)	1.06	1.06	1.06
Threonine^3)^ (%)	0.81	0.79	0.78

RSM, rapeseed meal.

1) Premix provided per kilogram of diet: Vit A 6,000 IU, thiamin 2.0 mg, riboflavin 4.0 mg, nicotinamide 42 mg, pyridoxine-HCl 4.0 mg, Vit B_12_ 0.01 mg, Vit D_3_ 2,000 IU, Vit E 30 IU, menadione 1.8 mg, calcium pantothenate 10.0 mg, biotin 0.15 mg, folic acid 0.85 mg, Fe (as ferrous sulfate) 80 mg, Cu (as copper sulfate) 8.0 mg, Mn (as manganese sulfate) 80 mg, Zn (as zinc sulfate) 65 mg, I (as potassium iodide) 0.50 mg, Se (as sodium selenite) 0.25 mg.

2) The crude protein content was a measured value, while the others were the calculated values.

3) The amino acids in diets refer to the true available amino acids.

**Table 2:** Primers sequences used in RT-PCR analysis

Genes	Primers (5′→3′)	Product size (bp)	Gene Bank^1)^
*Occludin*	Sense: GTTACTACTACAGCCCCTTGTTGG	142	NM_205128.1
	Antisense: AGCAGGATGACGATGAGGAA		
*Claudin-1*	Sense: AAGAAGATGCGGATGGCTGT	158	NM_001013611.2
	Antisense: AAGAGGGCTGATCCAAACTCAA		
*ZO-1*	Sense: CTTCAGGTGTTTCTCTTCCTCCTC	131	XM_015278980.2
	Antisense: CTGTGGTTTCATGGCTGGATC		
*β-actin*	Sense: ATTGTCCACCGCAAATGCTTC	113	NM_205518.1
	Antisense:AAATAAAGCCATGCCAATCTCGTC		

RT-PCR, real-time polymerase chain reaction; *ZO-1*, zonula occludens-1.

1) Genbank Accession Number.

**Table 3:** Effects of different levels of rapeseed meal substitution and glutamine supplementation on the growth performance of Qiandongnan Xiaoxiang chicken

Items	21 days of age			42 days of age		
	
	ADFI (g/d)	ADG (g/d)	FCR (g/d)	ADFI (g/d)	ADG (g/d)	FCR (g/d)
Control	15.55^ab^	6.27^bc^	2.48^bcd^	41.21^b^	12.99^b^	3.18^b^
10% RSM	14.58^cd^	5.65^d^	2.57^bc^	40.84^bc^	12.83^b^	3.19^b^
10% RSM+0.5%Gln	15.29^ab^	6.16^bc^	2.49^bcd^	41.62^ab^	13.32^ab^	3.14^b^
10% RSM+1.0%Gln	15.83^a^	6.70^a^	2.36^d^	43.99^a^	13.94^a^	3.16^b^
20% RSM	14.10^d^	5.13^e^	2.75^a^	38.22^d^	11.50^c^	3.33^a^
20% RSM+0.5%Gln	15.24^abc^	5.87^cd^	2.61^ab^	38.71^cd^	12.05^c^	3.21^ab^
20% RSM+1.0%Gln	15.45^ab^	6.38^ab^	2.43^cd^	41.16^b^	12.95^b^	3.18^b^
SEM	0.126	0.097	0.029	0.417	0.154	0.016
p-value	<0.001	<0.001	0.002	0.001	<0.001	0.046
Main effect						
RSM						
10%	15.24	6.17^x^	2.48^y^	42.15^x^	13.36^x^	3.16^y^
20%	14.93	5.79^y^	2.60^x^	39.36^y^	12.17^y^	3.24^x^
Gln						
0%	14.83^z^	5.39^y^	2.67^y^	39.53^y^	12.16^y^	3.26^x^
0.5%	15.20^y^	5.93^x^	2.56^y^	40.22^y^	12.58^y^	3.18^y^
1%	15.45^x^	6.45^x^	2.40^x^	42.11^x^	13.25^x^	3.17^y^
p-values						
RSM	0.068	0.004	0.015	<0.001	<0.001	0.006
Gln	<0.001	<0.001	0.003	0.001	<0.001	0.023
RSM×Gln	0.540	0.684	0.642	0.981	0.726	0.205

Values are mean and standard error of the mean, n = 10 per treatment group.

ADFI, average daily feed intake; ADG, average daily weight gain; FCR, feed conversion ratio; RSM, rapeseed meal; Gln, Glutamine.

Broilers in the control group were fed with basal diet. Broilers in 10% and 20% RSM were fed with diet containing 10% or 20% RSM, respectively. 10% RSM+0.5% Gln, 10% RSM+1.0% Gln 20% RSM+0.5% Gln, and 20% RSM+1.0% Gln means that 0.5% Gln or 1% Gln was separately supplemented based on 10% and 20% RSM inclusion.

In the same array, values with different small letters superscripts mean significant difference (p<0.05), while with the same or no letter superscript mean no significant difference (p>0.05).

a–e Superscripts for means belong to one-way analysis of variance analysis among 7 treatment groups.

x–z superscripts for means belong to 2×3 factorial analysis (except for the control group).

**Table 4:** Effects of different levels of rapeseed meal substitution and glutamine supplementation on the contents of total protein and glucose of Qiandongnan Xiaoxiang chicken

Items	21 days of age		42 days of age	
	
	TP (g/L)	Glu (g/L)	TP (g/L)	Glu (g/L)
Control	22.68^bc^	13.08	27.61^bcd^	11.60
10% RSM	20.82^cd^	12.77	27.01^cd^	11.52
10% RSM+0.5%Gln	22.92^ab^	12.87	29.11^b^	11.64
10% RSM+1.0%Gln	24.81^a^	13.02	31.24^a^	12.07
20% RSM	18.22^e^	12.04	24.69^e^	11.49
20% RSM+0.5%Gln	19.86^de^	12.96	26.02^de^	12.30
20% RSM+1.0%Gln	23.34^ab^	12.91	28.24^bc^	12.07
SEM	0.384	0.236	0.356	0.121
p-value	<0.001	0.765	<0.001	0.411
Main effect				
RSM				
10%	22.85^x^	12.89	29.12^x^	11.74
20%	20.48^y^	12.64	26.32^y^	11.95
Gln				
0%	19.52^z^	12.40	25.85^z^	11.50
0.5%	21.39^y^	12.92	27.57^y^	11.97
1%	24.08^x^	12.97	29.74^x^	12.07
p-values				
RSM	<0.001	0.554	<0.001	0.452
Gln	<0.001	0.488	<0.001	0.204
RSM×Gln	0.501	0.706	0.802	0.505

TP, total protein; Glu, glucose.

Values are mean and standard error of the mean, n = 10 per treatment group.

Broilers in the control group were fed with basal diet. Broilers in 10% and 20% RSM were fed with diet containing 10% or 20% RSM, respectively. 10% RSM+0.5% Gln, 10% RSM+1.0% Gln 20% RSM+0.5% Gln and 20% RSM+1.0% Gln means that 0.5% Gln or 1% Gln was separately supplemented based on 10% and 20% RSM inclusion.

In the same array, values with different small letters superscripts mean significant difference (p<0.05), while with the same or no letter superscript mean no significant difference (p>0.05).

a–e Superscripts for means belong to one-way analysis of variance analysis among 7 treatment groups.

x–z Superscripts for means belong to 2×3 factorial analysis (except for the control group).
