# Supplementary material for: Effects of substitution of soybean meal with rapeseed meal and glutamine supplementation on growth performance, intestinal morphology, and intestinal mucosa barrier of Qiandongnan Xiaoxiang Chicken
Source: Anim Biosci. 2022 Sep 15;35(11):1711–24. doi: 10.5713/ab.21.0467 (PMC9659468; doi:10.5713/ab.21.0467)
Supplement: Supplementary file 1 [file ab-21-0467-suppl.pdf]

March 21, 2022

Dear editor and reviewers,

Thank you for your letter of Editor Decision and for the reviewers' comments concerning our manuscript of **AB-21-0467** entitled "**Effects of substitution of soybean meal with rapeseed meal and glutamine supplementation on growth performance, intestinal morphology and intestinal mucosa barrier of Qiandongnan Xiaoxiang Chicken**". Thank you very much for your patience, and we appreciate the referees for their nice comments on our manuscript. We have studied their comments carefully and revised our manuscript according to reviewers` comments. Revised parts are showed in red font in the revised manuscript. The line numbers refers to our new revised manuscript. Our responses to the reviewers' comments are as follow.

Besides, the information of "‡College of Animal Science and Technology, Jilin Agricultural University, No. 2888, Xincheng Road, Jingyue District, Changchun 130118, People's Republic of China." was removed in the revised manuscript.

Hopefully we have addressed all of your concerns.

Thank you once again.

Sincerely,

B. L. Zhang

Zunyi Normal College

E-mail address: bolin-zhang@163.com

Reviewer 1

Comment 1: Line 47 says “inhibitory effects”; it should say “negative effects”.

Response: Dear reviewer, thanks for your good advice. We have replaced “inhibitor effects” with “**negative effects**” in **Line 45** in the revised manuscript. Besides, “improve” was replaced with “**reverse**” in **Line 45** in the revised manuscript. The revision was marked in red.

Comment 2: Lines 57 to 61. Change to present tense.

Response: Dear reviewer, thanks for your suggestion. The word “was” was revised as “**is**” in **Line 53 and Line 54** in the revised manuscript. Besides, “could” was replaced with “**can**” and “limited” was replaced with “**limit**” in **Line 55 and Line 57** in the revised manuscript. “evidence” was revised as “**evidences**” in **Line 57** in the revised manuscript. Thanks again.

Comment 3: Rewrite the sentence in line 62: it should be in the present tense.

Response: Dear reviewer, we have replaced “were” with “**are**” in **Line 58** in the revised manuscript. Moreover, “the results of which were confused” was revised as “**the results of which are inconsistent**” in **Line 58** in the revised manuscript.

Comment 4: It is not technically appropriate to state that “results are confused”. Rewrite clarifying your idea.

Response： Dear reviewer, thanks for your good advice. After careful consideration, “the results of which were confused” was replaced with “**the results of which are inconsistent**” in **Line 58** in the revised manuscript.

Comment 5: Rewrite the sentence in lines 63-64 considering that:

1) A study cannot “demonstrate” the absence of an effect. The absence of an effect cannot be statistically proven, but only an effect if it exists. Therefore, you can only state that “in a previous study no negative effect was detected” or “a previous study reported no negative effect”.

Response: Dear reviewer, thank you for your suggestion. The sentence “it has been demonstrated that the inclusion of RSM in diet up to 10% did not have any effects on …..” was revised as “**it was reported** that the inclusion of RSM in diet up to 10%

no negative effect on…". See details in **Lines 59-60** in the revised manuscript.

2) "Did not have any adverse effect" is literally impossible to be statistically proven. Hypothesis tests can only detect differences if the null hypothesis is rejected, but can never reject the alternate hypothesis to state that the null is true.

Response: Dear reviewer, we had modified our sentence according to your suggestion. See details in **Lines 59-60** in the revised manuscript. Thanks again.

Comment 6: Please carefully check the whole manuscript considering the criteria in the previous comment.

Response: Dear reviewer, according to your suggestion, we have check the whole manuscript carefully. The revisions are as follows:

(1) "was" was replaced with "**is**" in **Lines 340-341** in the revised manuscript.

(2) "were" was replaced with "**are**" in **Lines 364-365** in the revised manuscript.

(3) "played" was replaced with "**play**" in **Line 366** in the revised manuscript.

(4) "served" was replaced with "**serve**" in **Line 369** in the revised manuscript.

(5) "Chicken" was replaced with "**chicken**" in **Line 4, 23, 29, 48, 100, 218, 241, 309, 311, 339, 375, 391,392, 394, 564, 575, 585 and 596** in the revised manuscript.

(6) "chickens" was replaced with "**chicken**" in **Line 103.** And **"chickens"** was replaced with **"Chicken" in Line 523, 536, 548, 551, 554 and 558** in the revised manuscript.

(7) "day" was revised as "**days**" in **Line 33, 36, 37, 40, 41, 182, 183, 184, 186, 187, 190, 191, 193, 194, 196, 201, 203, 205, 207, 208, 212, 213, 214, 218, 219, 220, 223, 227, 231, 233, 238, 241, 243, 244, 245, 247, 249, 254, 257, 259, 260, 264, 280, 283, 285, 310, 313, 319, 333, 345, 346, 373, 375, 395, 564, 575, 585 and 596** in the revised manuscript.

Comment 7: Avoid using the term "significant" to qualify the relevancy or size of an effect, but only to refer to statistically significant results. Therefore, to avoid confusion, always show the P value within parenthesis, or avoid using that word, whatever works best for you. Ejm: Line 66.

Response: Dear reviewer, thank you for your good advice. We have deleted "significant" according to your suggestion. See details in **Line 61** in the revised manuscript. Besides, "significantly" is also deleted in **Line 62** in the revised manuscript. "0-21 days" and

"response" were replaced with "**1-21 days**" and "**responses**" in **Line 63**, respectively.

Comment 8: Line 65: "suggested" does not work with "resulted in a decrease", because the result of a study is not a possibility. However, "suggested" works with "results in a decrease", because that is a generalization. Check these criteria in the whole manuscript.

Response: Dear reviewer, Thanks for your suggestion. "the inclusion of ….1-21 days" in Lines 61-62 in the revised manuscript descripted the experimental results obtained from a previous study conducted by McNeill et al. Because the experiment has been completed, it may be appropriate to use the past tense to describe its relative results. After careful consideration, "suggested" was replaced with "**found**" in **Line 61** in the revised manuscript.

Besides, "demonstrated" was replaced with "**reported**" in **Line 68** in the revised manuscript, "demonstrated" was replaced with "**found**" in **Line 71** in the revised manuscript. "illustrated" was revised as "**found**" in **Line 83** in the revised manuscript. "demonstrated" and "suggested" were replaced with "**reported**" and "**found**" in Line **302** and **Line 303** in the revised manuscript, respectively. "resulted" was revised as "**results**" in **Line 307**, "demonstrated" was replaced with "**found**" in **Line 316**, "was" was replaced with "**is**" in **Line 322**, "demonstrated" was revised as "**reported**" in **Line 323**, "demonstrated" was revised as "**found**" in **Line 331**, "indicated" was replaced with "**found**" in **Line 346**, "could" was revised as "**can**" in **Line 359**, "promoted" was revised as "**promote**" in **Line 360** in the revised manuscript.

Comment 9: Line 73 should say "to consider".

Response: Dear reviewer, thanks for your suggestion. "for considering" was revised as "**to consider**" in **Line 68** in the revised manuscript according to your suggestion. Thank you again.

Comment 10: The English language is grammatically correct, but the general ideas are lost in many sentences. A technical writing review is highly encouraged.

Response: Dear reviewer, thanks for your suggestion. After careful checking the whole manuscript, we made some modifications as follows:

(1) We "resulted in the decreased" was revised as "**decreased**" in **Line 70** in the revised manuscript.
(2) "was" was deleted in **Line 72** in the revised manuscript.
(3) "the broilers" was revised as "**the broiler**" in **Line 76** in the revised manuscript.
(4) "of" and "the duodenum" were separately replaced with "**in the**" and "**duodenum**" in **Line 84** in the revised manuscript.
(5) "maintenance" was revised as "**maintain**" in **Line 86** in the revised manuscript.
(6) "was" was revised as "**has been**" in **Line 87** in the revised manuscript.
(7) "literature" was revised as "**literatures**" in **Line 88** in the revised manuscript.
(8) "level" was revised as "**levels**" in **Line 92** in the revised manuscript.
(9) "was" was revised as "**were**" in **Line 97** in the revised manuscript.
(10) "were" was revised as "**are**" in **Line 109** in the revised manuscript.
(11) "the" was revised as "**a**" in Line **111** in the revised manuscript.
(12) "is" and "varies" were replaced with "**was**" and "**varied**" in **Line 113** and **Line 114** in the revised manuscript, respectively.
(13) "**,**" was added in **Line 121** in the revised manuscript.
(14) "**the**" was added in **Line 128** in the revised manuscript.
(15) "21" was revised as "**21 d**" in **Line 134** in the revised manuscript.
(16) "respectively" was replaced with "**separately**" in **Line 136** in the revised manuscript.
(17) "isolation" was revised as "**extracted**" in **Line 155** in the revised manuscript.
(18) "OD260/OD280" was replaced with "**OD_{260}/OD_{280}**" in **Line 157** in the revised manuscript.
(19) "**calculated**" was added in **Line 164** in the revised manuscript.
(20) "broilers" was revised as "**broiler**" in **Line 173** in the revised manuscript.
(21) "and" was revised as "**or**" in **Line 184** in the revised manuscript.
(22) "**diet**" was added in **Line 184, 227, 233, 267, 270, 271, 275, 278, 291, 292, 294, 296,** and **298** in the revised manuscript.
(23) "**inclusion**" was added in **Line 181, 186, 191, 196, 202, 204, 206, 216, 221, 228, 232, 240, 244, 261, 264 and 286** in the revised manuscript.
(24) "and" was replaced with "**or**" in **Line 184 and 195**.

(25) "**diet containing**" was added in **Line 223** in the revised manuscript.

(26) "the diet containing the inclusion of 20% RSM" was replaced with "**diet containing 20% RSM**" in **Line 272** in the revised manuscript.

(27) "**group**" was added in **Line 289** in the revised manuscript.

(28) "the diets" was revised as "**with diets**" in **Line 335** in the revised manuscript.

(29) "**the**" was added in **Line 310, 337, and 363** in the revised manuscript.

(30) "**the**" was deleted in **Line 333** in the revised manuscript.

(31) "of" was deleted in **Line 325** in the revised manuscript.

(32) "nutrients metabolism" was revised as "**nutrition metabolism**" in **Line 338** in the revised manuscript.

(33) "effector factor" was revised as "**effector**" in **Line 375** in the revised manuscript.

(34) "the diet" was revised as "**diet**" in **Line 33**9 in the revised manuscript.

(35) "after Gln supplementation" was revised as "**by Gln addition**" in **Line 381** in the revised manuscript.

(36) "supplementation" was deleted in **Line 395** in the revised manuscript. Besides, "amelioration" was revised as "**ameliorated**" in **Line 396** in the revised manuscript.

(37) "diets" was revised as "**diet**" in **Line 530 and 543** in the revised manuscript, and "inclusion" was added in **Line 532 and 545** in the revised manuscript.

(38) "diets" was replaced with "**diet**" in **Line 566, 567, 577, 578, 587, 588, 598 and 599**, and "addition" was replaced with "**inclusion**" in **Line 569, 580, 590 and 601** in the revised manuscript.

Comment 11: Line 79 should say "the small intestine is".

Response: Dear reviewer, thank you for your good advice. We have revised it according to your suggestion. See details in **Line 74** in the revised manuscript. Thanks again.

Comment 12: Past tense should be used as a general rule for most of the manuscript when explaining what was done in the study and what was found, but not in the Introduction when talking about information that is (not was) available.

Response: Dear reviewer, thank you for your suggestion. After careful check, we revised "intestinal utilizes" as "**intestine can utilize**" in **Line 82** and revised "was" as "**has been**" in **Line 87** in the revised manuscript.

Comment 13: A connector is missing between "functions" and "the sooner it achieves" in line 81.

Response: Dear reviewer, we are sorry for this mistake. We have revised the sentence according to your suggestion. "**Therefore**" was added in **Line 75** in the revised manuscript. And the revision was marked in red.

Comment 14: Line 83: "conduct nutritional modulation"? Clarify the idea.

Response: Dear reviewer, thank you for your good advice. After careful consideration, "**such as exogenous active substances addition,**" was added in **Lines 78-79** in the revised manuscript. Thank you again.

Comment 15: Units in lines 116-117 are not shown.

Response: Dear reviewer, we have added units "**cm**" in **Line110** in the revised manuscript. In **Line111 and 112** in the revised manuscript, we also added "**°C**". Thanks again.

Comment 16: Line 148 should say "Intestinal morphology measurements"

Response: Dear reviewer, thanks for your good advice. We have replaced **"Intestine morphology measurement"** with "**Intestinal morphology measurements**" in **Line 144** in the revised manuscript.

Comment 17: Line 149 "for morphological…." Something is missing there.

Response: Dear reviewer, we are sorry for this mistake. After careful check, we have revised "morphological" as "**morphological measurements**" in **Line 145** in the revised manuscript. Thank you.

Comment 18: Line 107: replace "native" with something more appropriate.

Response: Dear reviewer, we have replaced "native" with "**newly-hatched**" in **Line 102** in the revised manuscript.

Comment 19: Line 175. Should say "consisted of the effects of RSM inclusion (10%"

Response: Dear reviewer, thank you for your suggestion. We have revised "consisted of the effects of diet (10% or 20% RSM)" as "**consisted of the effects of RSM inclusion (10% or 20%)** in **Line 171** in the revised manuscript.

Comment 20: Line 176 should say "for growth performance". Never use "parameter" to denote a "response variable".

Response: Dear reviewer, we have replaced "the parameter of growth performance" with "**for growth performance**" according to your suggestion. See details in **Line 172** in the revised manuscript.

Comment 21: Table 1: should say "Basal diet" (remove "the")

Response: Dear reviewer, thanks for your suggestion. In the revised manuscript, "the basal diet" was revised as **"Basal diet"** in **Table 1** according to your suggestion.

Comment 22: Table 1: Specify if amino acid values are total or digestible.

Response： Dear reviewer, thank you for your suggestion. Diets used in this experiment were formulated to meet or exceed the standard of nutrient requirement of yellow chickens. Besides, the amino acids in diets refer to the true available amino acids. We have added this information in the revised manuscript. See details in **Line 518** in the revised manuscript.
